# Your emotion or mine: labeling feelings alters emotional face perception—an ERP study on automatic and intentional affect labeling

**DOI:** 10.3389/fnhum.2013.00378

**Published:** 2013-07-23

**Authors:** Cornelia Herbert, Anca Sfärlea, Terry Blumenthal

**Affiliations:** ^1^Department of Psychology, University of WürzburgWürzburg, Germany; ^2^Department of Psychology, Wake Forest UniversityWinston-Salem, NC, USA

**Keywords:** emotion regulation, language-as-context, affect labeling, face processing, event-related brain potentials, social context, social cognition, perspective taking

## Abstract

Empirical evidence suggests that words are powerful regulators of emotion processing. Although a number of studies have used words as contextual cues for emotion processing, the role of *what* is being labeled by the words (i.e., one's own emotion as compared to the emotion expressed by the sender) is poorly understood. The present study reports results from two experiments which used ERP methodology to evaluate the impact of emotional faces and self- vs. sender-related emotional pronoun-noun pairs (e.g., my fear vs. his fear) as cues for emotional face processing. The influence of self- and sender-related cues on the processing of fearful, angry and happy faces was investigated in two contexts: an automatic (experiment 1) and intentional affect labeling task (experiment 2), along with control conditions of passive face processing. ERP patterns varied as a function of the label's reference (self vs. sender) and the intentionality of the labeling task (experiment 1 vs. experiment 2). In experiment 1, self-related labels increased the motivational relevance of the emotional faces in the time-window of the EPN component. Processing of sender-related labels improved emotion recognition specifically for fearful faces in the N170 time-window. Spontaneous processing of affective labels modulated later stages of face processing as well. Amplitudes of the late positive potential (LPP) were reduced for fearful, happy, and angry faces relative to the control condition of passive viewing. During intentional regulation (experiment 2) amplitudes of the LPP were enhanced for emotional faces when subjects used the self-related emotion labels to label their own emotion during face processing, and they rated the faces as higher in arousal than the emotional faces that had been presented in the “label sender's emotion” condition or the passive viewing condition. The present results argue in favor of a differentiated view of language-as-context for emotion processing.

## Introduction

Emotion perception in oneself and others is an important aspect of successful social interaction. It is important for emotional self-regulation, and is often compromised in affective and mental disorders such as autism, sociopathy, schizophrenia, and depression, as well as in disorders associated with emotional blindness (alexithymia).

Narrative writing has been shown to have positive effects on emotional self-regulation (Hayes and Feldman, 2004) and individual well-being, possibly by increasing self-referential processing and reappraisal of emotionally challenging events from different perspectives (Seih et al., 2011; for an overview: Pennebaker and Chung, 2011). There is strong evidence from cognitive emotion regulation research supporting reappraisal as one of the most effective cognitive strategies for intentional down-regulation of negative feelings experienced in real life situations, or in the laboratory during viewing of emotion inducing stimuli including pictures, faces, or films (Gross, 2002; John and Gross, 2004; Blechert et al., 2012). Similarly, several studies investigating the neural correlates of self-referential processing suggest that appraising emotional stimuli in terms of their personal relevance can lead to adaptive emotion processing (e.g., Ochsner et al., 2004; Northoff et al., 2006; Moran et al., 2009).

An important key component of narrative writing is affect labeling. During narrative writing people learn to “put their feelings into words” and to express or reframe them verbally. The success of narrative writing suggests that words can be powerful regulators of emotions. Empirical support for this suggestion comes from neurophysiological research investigating brain responses, in addition to subjective indicators of emotion processing in participants exposed to affective labels or verbal descriptions while viewing emotional stimuli. For example, Foti and Hajcak (2008), and Macnamara et al. (2009) recorded event-related brain potentials (ERPs) from the electroencephalogram (EEG). Participants viewed unpleasant pictures which were preceded by neutral or negative verbal sentences. Processing of neutrally framed unpleasant pictures decreased ratings of picture emotionality, self-reported negative affect, and amplitudes of the late positive potential (LPP). The LPP often shows larger amplitudes during processing of emotional stimuli than during the processing of neutral stimuli (Olofsson et al., 2008). Attenuated LPP amplitudes to verbally framed unpleasant pictures therefore suggest a decrease in the depth of emotional stimulus encoding.

Verbal reframing effects are not restricted to neutral cues or sentences. Hariri et al. (2000) and Lieberman et al. (2007) scanned brain activity patterns by means of functional imaging while individuals viewed faces and affective labels. Affective labels consisted of simple words which were presented together with unpleasant faces or socially stereotyped faces, including black and white people. The task was to indicate which of the words fit best with the emotion depicted in the face. Control conditions included passive stimulus viewing (without labels), non-verbal affect matching (i.e., verbal labels were replaced by faces), and shape matching of simple geometric figures. Affect labeling with words as cues was the only condition that decreased amygdala activation significantly. It also enhanced activity in the right ventrolateral cortex (Lieberman et al., 2007), an important prefrontal control area involved in a variety of tasks requiring executive control of attention, response-inhibition, and intentional emotion regulation (Cohen et al., 2013). Consistent with these neurophysiological observations affect labeling with words as cues decreased peripheral-physiologic responses of emotional arousal and, in another study, reduced self-reported negative affect and distress in response to unpleasant emotional pictures to a similar extent as did intentional emotion regulation by means of reappraisal (Lieberman et al., 2011). Along these lines, developmental research has demonstrated a relationship between language impairments in childhood and diminished self-control (Izard, 2001), and poor emotional competence and emotion regulation abilities during adulthood (Fujiki et al., 2004). Furthermore, reducing the accessibility of emotion words experimentally (via a semantic satiation procedure) has been shown to decrease emotion recognition accuracy during face processing in healthy subjects (Lindquist et al., 2006; Gendron et al., 2012), a finding that matches with clinical observations of decreased face recognition abilities in patients with aphasia, who experience extreme difficulties in naming words (Katz, 1980).

Together, all these findings support the theoretical view that language provides a conceptual context for emotion processing (Barrett et al., 2007, 2011). Specifically, they suggest that words as affective labels can improve emotion recognition and at the same time regulate emotion processing much like intentional emotion regulation strategies, lending support for the idea of an incidental emotion regulation process underlying language processing in emotional contexts (Lieberman et al., 2011). Therefore, affect labeling has been suggested as an additional technique for emotion regulation in clinical and therapeutic settings (Tabibnia et al., 2008; Lieberman, 2011; Kircanski et al., 2012), especially in patients who, due to the severity of their symptoms, are less sensitive to more complex cognitive behavioral interventions that often require that people are able and willing to reflect in detail upon their feelings and the logic of their maladaptive appraisals.

Two questions have not yet been answered: (1) are words (affective labels) equally effective in modulating emotion recognition and emotion processing when those labels directly relate to the participants' own emotion as compared to the emotion expressed in the sender's face? (2) would these effects be the same during intentional regulation as compared to automatic or uninstructed regulation? In other words, does it matter “what” is going to be labeled by the words (own emotion vs. emotion conveyed by the sender) and “how” (automatically or intentionally) this is done? One way to answer these questions would be to expose individuals to faces expressing discrete emotions, such as fear, anger, or happiness, and to instruct them to find words which best describe either their own emotions, or the emotion expressed by the sender's face. This would involve participants correctly identifying their own emotions and finding words to express them, and these abilities vary across individuals (Barrett et al., 2011). The appraisal of cortical and peripheral physiological changes associated with emotion processing are often limited to more basal emotional dimensions of perceived pleasantness (good-bad, like-dislike) or arousal intensity (calming-arousing). However, ERP methodology may be useful in identifying cue-driven emotion processing and intentional emotion regulation, using as affective cues pronoun-noun pairs that are either self-or sender-related.

Research on the processing of pronouns has shown that readers adopt a first person perspective (1PP) during reading of self-related pronouns and a third-person perspective (3PP) during reading of other-related pronouns (Borghi et al., 2004; Ruby and Decety, 2004; Brunye et al., 2009). Moreover, EEG studies have reported emotional pronoun-noun pairs describing the reader's own emotion (e.g., my fear, my fun) to be processed more deeply than pronoun-noun pairs making a reference to the emotion of others (e.g., his fear, his fun) or emotion words that contain no reference at all (e.g., the fun, the fear) (Herbert et al., 2011a,b). Further, in an imaging study, reading of self-related emotional pronoun-noun pairs selectively enhanced activity in medial prefrontal brain structures involved in the processing of one's own feelings (Herbert et al., 2011c), providing neurophysiological evidence that people spontaneously discriminate between the self and the other during reading (see also Walla et al., 2008; Zhou et al., 2010; Shi et al., 2011).

Self-related emotion labels (e.g., my fear, my happiness) provide a window to one's own emotions, linking one's own sensations with the emotion expressed by the sender's face. Sender-related labels (e.g., his/her fear, happiness) make a direct reference to the emotion expressed by the sender. Therefore, processing of self- and sender-related labels should have differential effects on how emotional information conveyed by a stimulus such as a face is decoded, and also which emotional reactions and feelings are experienced in return. While labels depicting one's own emotions should make facial expression more relevant to the self, thereby increasing attention capture by emotional faces, sender-related labels might specifically improve decoding of structural information from the face. As explained in more detail below, this should be accompanied by different modulation patterns of early brain potentials in the EEG, such as the face specific N170 and the early posterior negativity (EPN). In addition, self- and sender-related labels both contribute information that goes beyond the affective information available from the face. Both labels contain information required for appraising the meaning of the emotion expressed in the face. Making this information available to the subject could reduce processing resources required for appraising the emotional meaning of the faces. In the EEG, this should be reflected by modulation of late ERP components such as the LPP.

The aim of the present EEG-ERP study was to shed light on these questions by investigating how processing of self- and sender-related affective labels modulates emotional face processing across different stages of processing, i.e., from initial processing of emotional stimulus features to the more fine grained, in depth analysis of the emotional content of the presented faces. Two separate experiments were conducted. The first experiment investigated emotional face processing during an “unintentional labeling” task. This was done to investigate spontaneous effects of self- and sender-related affective labels on emotional face processing. The second experiment used an active emotion regulation context in which self- and sender-related affective labels served as cues for emotion regulation and faces as targets of emotion regulation. The active emotion regulation context was chosen to separate unintentional from intentional affect labeling processes, and to explore whether emotion regulation with affective labels and “unintentional” processing of affective labels would differentially modulate event-related brain potentials (ERPs) to emotional faces. Moreover, the active emotion regulation context will allow us to investigate whether emotion regulation with self- and sender-related affective labels will facilitate self-referential processing and cognitive reappraisal of emotional faces from different perspectives (1PP vs. 3PP). Specifically the active process of labeling and the intention to use these labels for emotion regulation should allow a person to get in touch with his/her feelings when self-related affective cues are being presented and to distance him- herself from the own feelings when faces are cued with sender-related affective cues.

By comparing ERPs elicited by the faces during the affective label conditions and during a control condition of passive face viewing it is possible to precisely determine the particular stages at which processing of self- and sender-related emotion labels impact emotional face processing, in which direction these effects will occur (up- vs. down-regulation), and if effects vary across the two processing conditions of spontaneous, unintentional processing (experiment 1) and active, intentional regulation (experiment 2). ERPs of interest included early and late ERP components, the P1, the face specific N170, the early posterior negativity potential (EPN), and the late positive potential (LPP) or slow wave (SW). These cortical components are thought to indicate stimulus-driven as well as sustained processing of emotional stimuli. The P1 reflects very early stimulus feature processing while the N170 reflects increased structural encoding and the EPN facilitated capture of attentional resources by stimuli of emotional relevance (Bentin and Deouell, 2000; Junghöfer et al., 2001; Schupp et al., 2004; Blau et al., 2007). Amplitude modulations of the LPP are thought to index sustained processing and encoding of emotional stimuli in functionally coupled, fronto-parietal brain networks (Moratti et al., 2011).

Furthermore, participants' subjective appraisals of the presented stimuli, their mood state, and their emotion perception and empathic abilities were assessed via self-report. As additional exploratory outcome measures, subjective measures could provide information about potential variables that mediate affect labeling and face processing.

## Methods: Experiment 1 and experiment 2

### Subjects

Twenty-one right-handed adults (16 females, 5 males), all native speakers of German, with a mean age of 22 years (*SD* = 3.1 years) participated in experiment 1. Seventeen right-handed adults (12 females, 5 males), all native speakers of German, with a mean age of 22 years (*SD* = 2.2 years) participated in experiment 2. Participants were recruited via the posting board of the University of Würzburg and received course credit or financial reimbursement of 15€ for participation. Exclusion criteria for participation were current or previous psychiatric, neurological, or somatic diseases, as well as medication for any of these. Participants reported normal audition, and normal or corrected to normal vision. Both experiments were conducted in accordance with the Declaration of Helsinki and methods were approved by the ethical committee of the German Psychological Society (http://www.dgps.de/en/).

Participants of experiment 1 and of experiment 2 had comparable scores on the Beck Depression Inventory (Hautzinger et al., 1994) (experiment 1: *M* = 5.3, *SD* = 3.1; experiment 2: *M* = 3.4, *SD* = 2.6). Both groups scored normally on the trait (experiment 1: *M* = 44.7, *SD* = 5.1; experiment 2: *M* = 45.5, *SD* = 4.3) and state (experiment 1: *M* = 39.6, *SD* = 4.5; experiment 2: *M* = 42.3, *SD* = 5.7) scales of the Spielberger State-Trait Anxiety Inventory (STAI, Laux et al., 1981). They reported more positive affect (experiment 1: *M* = 37.0, *SD* = 6.1; experiment 2: *M* = 38.8, *SD* = 5.9) than negative affect (experiment 1: *M* = 19.4, *SD* = 6.2; experiment 2: *M* = 17.8, *SD* = 4.7) on the PANAS mood assessment scales (Watson et al., 1988) and they did not differ in empathy (experiment 1: *M* = 14.19, *SD* = 2.2; experiment 2: *M* = 15.1, *SD* = 1.1), perspective taking (experiment 1: *M* = 14.3, *SD* = 2.3; experiment 2: *M* = 15, *SD* = 2.2), emotional intelligence (experiment 1: *M* = 117.9, *SD* = 7.1; experiment 2: *M* = 120.1, *SD* = 7.5), or emotional blindness (experiment 1: *M* = 50.6, *SD* = 6.7; experiment 2: *M* = 47.9, *SD* = 7.3). They did also not differ in self-esteem (experiment 1: *M* = 39.4, *SD* = 2.8; experiment 2: *M* = 39.2, *SD* = 3.1). Empathy and perspective taking were measured with the Saarbrückener Personality Questionnaire (Paulus, 2009), the German Version of the Trait Emotional Intelligence Questionnaire (TEIQue) was used for emotional intelligence (Freudenthaler et al., 2008) and emotional blindness was assessed with the German Version of the Toronto Alexithymia Scale (TAS-20, Bagby et al., 1994). Self-esteem was measured via the Frankfurter Self Concept Scale (FSSW, Deusinger, 1986). Habitual emotion regulation strategies including reappraisal or suppression were also assessed with a German translation of the emotion regulation questionnaire (ERQ, Gross and John, 2003). Again both groups reported comparable scores on the ERQ for using either reappraisal (experiment 1: *M* = 5.0, *SD* = 0.7; experiment 2: *M* = 4.7, *SD* = 1.2) or suppression (experiment 1: *M* = 3.6, *SD* = 1.0; experiment 2: *M* = 3.4, *SD* = 0.7) as an emotion regulation strategy in daily life.

### Stimuli (experiment 1 and experiment 2)

Faces (fearful, angry, happy, and neutral) were taken from the Karolinska Directed Emotional Face database (KDEF, Lundqvist et al., 1998). Affective labels were sixty pronoun-noun pairs. Twenty of these pairs were related to fear, 20 to anger, and 20 to happiness. Stimuli were presented in six randomized blocks, three self-related blocks and three sender-related blocks. Each block contained twenty faces (half male/half female characters) from one emotion category (fear, anger, or happiness) and twenty labels. Labels matched the emotion depicted in the face and were related to the participants' own emotions (e.g., my fear, my pleasure, my anger) in the self-related blocks and to the emotion of the sender's face (e.g., his/her fear, his/her pleasure, his/her anger) in the sender-related blocks. Thus, each block consisted of 20 emotion congruent trials. Labels and faces were presented for 1.5 s each, and separated by a fixation cross of 500 ms duration. Trials were separated by an inter-trial interval lasting about 1 s and blocks were separated by a fixation cross indicating the beginning of a new block.

**Figure 1:**
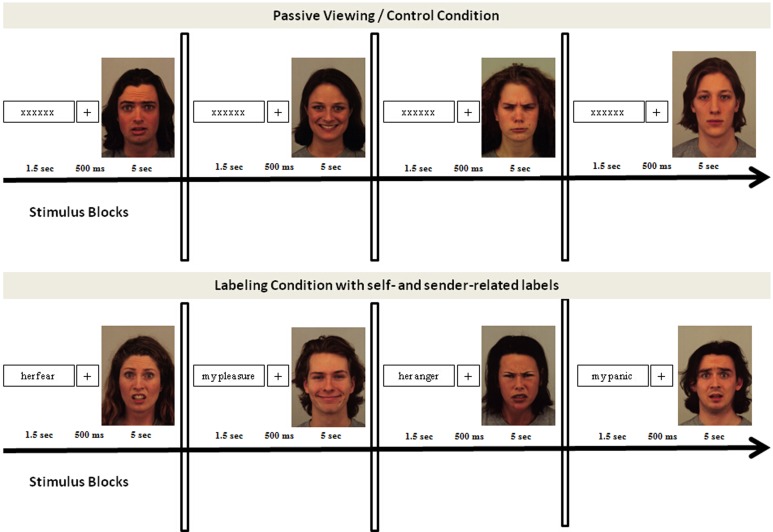
**Experimental set-up of experiment 1 and experiment 2**. Both experiments used the same design, consisting of a control condition and an affective label condition. The control condition was always presented first and consisted of blocks of fearful, happy, angry, and neutral faces. The affective label condition consisted of six blocks, three self-related and three sender-related blocks. Block order was randomized and stimuli were randomly assigned to the conditions.

Blocks of passive viewing, in which 20 faces (half male/half female characters) of each emotion category as well as 20 neutral faces were preceded by random letter strings instead of affective labels, served as control condition (see Figure 1). Akin to the experimental condition, each trial consisted of 1.5 s letter presentation, a 500 ms fixation-cross period, and a picture viewing period of 1.5 s. Trials were separated by inter-trial intervals of about 1 s and blocks by inter-block intervals. Block order was randomized. Emotional faces were randomly assigned to the blocks, such that none of the faces was repeated across blocks (self vs. sender) and conditions (control vs. affective label conditions). In line with previous studies, the passive viewing conditions were always presented first to guarantee a neutral baseline of emotional face processing. Also in line with previous studies, neutral faces were presented in the control condition, only (e.g., Hajcak and Nieuwenhuis, 2006; Hajcak et al., 2006; Blechert et al., 2012).

Affective labels and letter strings were presented in black letters (font “Times”; size = 40) centered on a white background of a 19 inch computer monitor. Faces were presented in color, centered on the computer screen. Stimuli were presented at a visual angle of 4°. Experimental runs were controlled by Presentation software (Neurobehavioral Systems Inc.). An overview of the experimental design is shown in Figure 1.

## Procedure

After arrival at the laboratory, participants were informed in detail about the EEG procedure; they were questioned about their handedness and health, and electrodes for EEG recording were attached before they received the following instructions.

### Experiment 1

In the control condition, participants were told to view the faces attentively without paying specific attention to the preceding letter strings. In the following “affective label” condition, they were told that again a series of faces would be presented, each of which would be preceded by a verbal cue describing either their own emotion to the face or the emotion of the person presented in the face. Participants were asked to attend to the stimulus pairs (cue and face), but received no instruction to appraise the stimuli in a specific way nor to intentionally regulate their emotions during face processing. Prior to the start of the experimental recording, participants were given practice trials to familiarize them with the task. After the experimental recording, participants were asked to rate the stimuli for valence and arousal on a nine-point paper-pencil version of the Self Assessment Manikin (Bradley and Lang, 1994), they were questioned about their experience during picture viewing, and they filled in the additional questionnaires for perspective taking, emphatic concerns, emotion perception abilities, and habitual emotion regulation strategies. Finally, they were debriefed in detail about the purpose of the experiment.

### Experiment 2

Experiment 2 used the same stimuli and experimental set-up as experiment 1. However, in experiment 2, participants were asked to control their emotions during face processing by means of the cues presented prior to each face. When cues were self-related to their own emotion they should try to use the cues to get in touch with their feelings and label them when looking at the faces (“label own emotion” blocks). When cues were related to the emotion of the sender's face (“label sender's emotion” blocks) they should try to use the cues to distance themselves from their own emotions by labeling the emotion of the sender's face when looking at the faces. Participants received practice trials prior to the start of the experimental recording sessions to ensure that they understood the task and to familiarize them with cue-driven intentional regulation. In addition, they were asked to indicate their regulation success as well as their present feelings immediately after each regulation block on nine-point Likert scales. After the experiment, they were questioned in more detail about their regulation experiences. They rated the stimuli for valence and arousal on the Self-Assessment Manikin (Bradley and Lang, 1994). Afterwards, they filled in the questionnaires on perspective taking, emphatic concerns, emotion perception and habitual emotion regulation strategies, and were debriefed in detail about the purpose of the experiment.

## Electrophysiological recordings (experiment 1 and experiment 2)

Electrophysiological data was recorded from 32 active electrodes using the actiCap system (Brain Products GmBH). For all electrodes impedance was kept below 10 kOhm. Raw EEG data were sampled at a rate of 500 Hz with FCz as reference. Off-line, EEG signals were digitally re-referenced to an average reference passing from 0.01 to 30 Hz, and corrected for eye-movement artifacts using the traditional algorithm by Gratton et al. (1983), implemented in the Analyzer2 software package (BrainProducts GMBH). Further artifacts due to head- or body-movements were rejected via a semi-automated artifact rejection algorithm. In total, this resulted in a loss of about 2–3 trails per block, leaving about 17 trials per block for averaging. Although signal to noise ratio increases with the number of averaged trials, recent research (Moran et al., 2013) has shown that differences in ERPs between experimental conditions can be reliably detected after a few averaged trials. This has been shown for late ERP components, which are more susceptible to background noise than early ERP components. Artifact-free EEG data were segmented from 500 ms before until 1500 ms after onset of the target faces using the 100 ms interval before target face onset for baseline correction. Baselines corrected epochs were then averaged for each experimental condition (affective label, control) and valence category (fear, anger, happy, neutral). Electrodes and time-windows for amplitude scoring of early (P1, N170, EPN) and late event-related brain potentials (LPP/slow wave) were determined in line with the previous literature on emotional face processing and by visual inspection of the grand mean average waveforms.

Amplitudes of early ERPs (P1, N170, and EPN) were analyzed at left and right posterior electrodes (O1, O2, PO10, PO9, P8, P7, TP10, TP9) from 80 to 120 ms (P1), from 140 to 180 ms (N170), and from 200 to 400 ms (EPN) post target face onset. Amplitudes of the late positive potential (LPP) were analyzed at parietal electrodes (P3, P4, Pz, P7, P8) in a time-window from 400 to 600 ms post target face onset. In experiment 2, intentional emotion regulation elicited a more pronounced cortical positivity (slow wave) over parietal electrodes compared to unintentional affect labeling. Akin to the LPP, amplitudes of this slow wave were analyzed at parietal electrodes (P3, P4, Pz, P7, P8), starting in a time-window from 400 to 800 ms from post-target face onset. ERP amplitudes were scored at each electrode as the averaged amplitude (in μV) in the respective time-window.

In addition, latencies of ERP amplitudes were defined via a semi-automatic peak detection algorithm of the Analyzer2 software package (BrainProducts GMBH). Latencies were analyzed in both experiments to determine if processing of affective labels had an influence on the speed of face processing.

Event-related potentials (P1, N1/EPN, and LPP) elicited during the processing of self- and sender-related affective labels were also analyzed from the epochs from 500 ms before until 1000 ms after word onset. The 100 ms interval before word onset was used for baseline correction. Amplitudes of early ERPs (P1, N1, and EPN) were analyzed at left and right posterior electrodes (O1, O2, PO10, PO9, P8, P7, TP10, TP9) from 80 to 120 ms (P1), 120 to 180 (N1), and from 200 to 400 ms (EPN) post word onset; amplitudes of the LPP were analyzed from 400 to 600 ms post word onset at the parietal electrodes P3, P4, and Pz.

## Data reduction and statistical analysis

### Electrophysiological data—experiment 1 and experiment 2

#### Affective labels

ERPs (P1, N1, EPN, LPP) elicited during the presentation of affective labels were analyzed with repeated measures analyses of variance (ANOVAs), which contained the factors *emotion (fearful, happy, angry)*, *label (self vs. sender)*, and *electrode location* as within-subject factors.

#### Faces

For faces the ANOVAs contained the factors *emotion (fearful, happy, angry)*, *condition (self vs. sender vs. passive viewing)*, and *electrode location* as within-subject factors. We also evaluated whether processing of emotional faces elicited larger ERP amplitudes than processing of neutral faces during passive viewing. The ANOVAs for the passive viewing comparisons included the factors *valence* (*fearful, happy, angry and neutral*) and *electrode location* as within-subject factors and were conducted for each ERP component of interest (P1, N170, EPN, and LPP/slow wave).

Where appropriate, p-values were adjusted according to Greenhouse and Geisser (1959). Significant main effects were decomposed by simple contrast test and results from these comparisons are reported uncorrected at *p* < 0.05. Interactions were followed up with planned comparisons within a row or column of the design matrix, to decrease the total number of comparisons by avoiding those that would not make theoretical sense. For example, an interaction between *condition* and *emotion* might involve contrasting fearful_self_ vs. fearful_other_, but would not involve a contrast between fearful_self_ vs. angry_other_, since this would involve a confound across levels of both variables. Again, results are reported *p* < 0.05, uncorrected.

## Behavioral data – experiment 1 and experiment 2

### Subjective ratings

Ratings were analyzed with repeated measures analyses of variance (ANOVAs). For faces, the ANOVAs contained the factors *emotion* (*fearful, happy, angry*) and *condition (passive viewing vs. self vs. sender)* as within-subject factors. Similar to the analysis of the ERPs, separate ANOVAs were calculated to consider differences in ratings between emotional and neutral faces. Ratings of the affective labels were analyzed with ANOVAs containing the factors *emotion* (*fearful, happy, and angry*) and *label (self vs. sender)* as within-subject factors. Self-report data including reports about changes in mood and regulation success given after each regulation block were also analyzed in separate ANOVAs, each containing the factors *emotion* (*fearful, happy, and angry*) and *label (self vs. sender)* as within-subject factors.

### Correlational analysis: ERPs and self-report measures

In both experiments, ERPs (P1, N170, EPN, and LPP/slow wave) were correlated with participants' self-report measures. Self-report measures of interest included positive and negative affect, depression, state and trait anxiety, and empathic concerns, perspective taking, self-esteem, and the ability or inability to describe and identify feelings as measured with the subscales of the Toronto Alexithymia Scale and the TEIQue emotional intelligence questionnaire. Although these analyses are exploratory in the present study, a mediating role of these variables could theoretically be expected based on clinical findings and the literature on individual differences (e.g., Herbert et al., 2011a,d; Moratti et al., 2011).

## Results

### Experiment 1—labeling without intentional regulation instruction

#### Electrophysiological data

***Affective Labels***. Processing of self- and sender-related labels did not differ in the P1, N1, and EPN time-windows. In the time-window of the LPP, amplitudes were more pronounced for self-related than for sender-related affective labels. The main factor *label* was significant, *F*_(1, 20)_ = 7.08, *p* = 0.02.

***Faces***. *Emotional faces: self vs. sender vs. control (passive viewing).* Emotional face processing differed significantly when preceded by affective labels as compared to during passive viewing. A first difference was observed in the P1 time-window and is indicated by the main factor *condition*, *F*_(2, 40)_ = 4.0, *p* = 0.027. P1 amplitudes were significantly more positive for fearful, angry, and happy faces during the “affective label” conditions compared to during passive viewing. During passive viewing, P1 amplitudes did not differ significantly between fearful, angry, happy, and neutral faces.

N170 amplitudes showed a main effect of *emotion*, *F*_(2, 40)_ = 6.4, *p* = 0.003: Fearful faces elicited significantly larger N170 amplitudes than angry faces, *F*_(1, 20)_ = 14.1, *p* = 0.001. In addition, the interaction of the factors *emotion* and *condition* was significant, *F*_(4, 80)_ = 3.0, *p* = 0.05: fearful faces elicited significantly larger N170 amplitudes when preceded by labels describing the sender's emotion as compared to when preceded by labels describing the viewer's own emotion, *F*_(1, 20)_ = 4.1, *p* = 0.05, as well as compared to when presented without any labels (control condition), *F*_(1, 20)_ = 12.8, *p* = 0.002. For emotional faces preceded by labels describing the viewer's own emotion, N170 amplitudes did not differ from passive viewing of emotional faces. During passive viewing, the factor *valence* was also significant, *F*_(3, 60)_ = 4.64, *p* = 0.02: fearful faces elicited significantly larger N170 effects in comparison to neutral faces, *F*_(1, 20)_ = 7.03, *p* = 0.02.

EPN amplitudes showed a significant main effect of *condition*, *F*_(2, 40)_ = 3.6, *p* = 0.05. EPN amplitudes were significantly more pronounced for fearful, angry, and happy faces when they were preceded by self-related labels than when they were presented without any labels in the control condition, *F*_(1, 20)_ = 4.48, *p* = 0.046. Cueing fearful, angry, and happy faces with emotion words describing the emotion of the sender's face did not change EPN amplitudes relative to when presented without any labels (control condition). EPN amplitudes did also not differ significantly between emotional and neutral faces during passive viewing. The factor *valence* was not significant, *F*_(3, 60)_ = 1.2, *p* = 0.32.

The amplitude of the LPP was significantly modulated by the factor *condition*, *F*_(2, 40)_ = 3.8, *p* = 0.05. LPP amplitudes were attenuated for emotional faces regardless of their valence (fearful, angry, and happy) when preceded by self-related and sender-related affective labels as compared to when preceded by letter strings (control condition). During the control condition, fearful, *F*_(1, 20)_ = 14.3, *p* = 0.001, as well as happy faces, *F*_(1, 20)_ = 11.5, *p* = 0.002, elicited significantly larger LPP amplitudes compared to neutral faces. The factor *valence* was significant, *F*_(3, 60)_ = 4.86, *p* = 0.004.

In contrast to amplitude measures, cueing faces with affective labels had no significant effects on ERP latencies.

**Figure 2:**
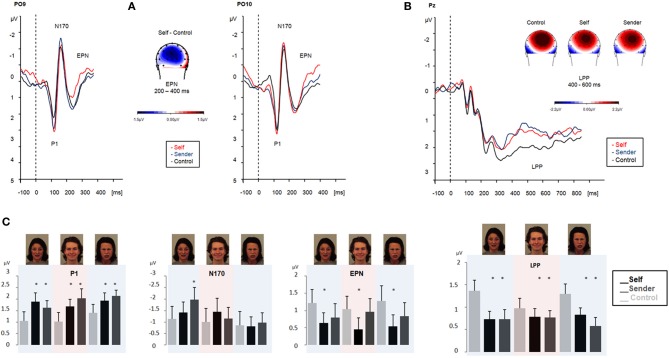
**ERPs obtained during emotional face processing in experiment 1**. ERPs are collapsed across angry, fearful, and happy faces and contrasted with passive viewing of emotional faces (black line). Upper left panel **(A)** displays ERP modulation patterns in the early time-windows (P1, N170, and EPN, respectively). Upper right panel **(B)** displays amplitudes in the LPP time-window. Maps display the topographic distribution of the ERP patterns in μV. Lower panel **(C)** overview of P1, N170, EPN and LPP modulation (means and SEMs) during emotional face processing in experiment 1. Color shadings highlight ERP modulation for each emotional category (fearful, angry, and happy).

ERP results are summarized in Figure 2.

#### Behavioral data

***Ratings***. Fearful and angry faces were rated as significantly more negative in valence compared to happy faces, regardless of whether faces were cued with affective labels or not, *F*_(2, 40)_ = 201.9, *p* < 0.01. Ratings of emotional faces did not differ in terms of arousal, *F*_(2, 40)_ = 2.7, *p* = 0.08, but emotional faces were rated as higher in arousal than neutral faces. This was true for faces shown in the control condition, *F*_(3, 60)_ = 26.7, *p* < 0.01, and for faces shown in the “affective label” conditions, *self*: *F*_(3, 60)_ = 26.2, *p* < 0.01, and *sender*: *F*_(3, 60)_ = 29.9, *p* < 0.01.

Self-related labels were rated as higher in arousal and as more relevant to the self compared to sender-related labels, *arousal: F*_(1, 20)_ = 11.1, *p* < 0.01, *self-relevance*: *F*_(1, 20)_ = 37.0, *p* < 0.01. This self-relevance effect was most pronounced for positive pronoun-noun pairs, *F*_(2, 40)_ = 10.2, *p* < 0.01. Valence ratings confirmed a self positivity bias, *F*_(2, 40)_ = 5.1, *p* = 0.03. Positive pronoun-noun pairs were rated more positive when related to the self.

Rating data are summarized in Table 1 and Table 2.

**Table 1:** **Experiment 1: rating data of emotional faces which were preceded by either self-related pronoun-noun pairs, sender-related pronoun-noun pairs, or no pronoun-noun pairs (control condition)**.

	**Fear**	**Anger**	**Happiness**	**Neutral**
**SELF-RELATED**				
Valence	3.40 (0.48)	3.01 (0.52)	6.69 (0.95)	
Arousal	4.83 (1.59)	4.99 (1.57)	4.43 (1.37)	
**SENDER-RELATED**				
Valence	3.27 (0.65)	3.04 (0.73)	6.78 (0.82)	
Arousal	4.89 (1.67)	4.91 (1.53)	4.29 (1.39)	
**CONTROLS**				
Valence	3.41 (0.59)	2.99 (0.61)	6.57 (0.77)	4.67 (0.46)
Arousal	4.68 (1.55)	4.93 (1.76)	4.21 (1.36)	2.69 (1.41)

Scales ranged from 1 (extremely negative valence, extremely low arousal) to 9 (extremely positive valence, extremely high arousal). Standard deviations are in parentheses.

**Table 2:** **Experiment1: mean valence, arousal, and self-relevance ratings of self-related and sender-related pronoun-noun pairs obtained after the experimental recordings**.

	**Fear**	**Anger**	**Happiness**
**SELF-RELATED PRONOUN-NOUN PAIRS**			
Valence	3.10 (0.95)	3.21 (1.04)	7.42 (0.99)
Arousal	5.02 (1.80)	4.77 (1.69)	5.62 (1.36)
Self-Relevance	5.54 (1.66)	5.11 (1.35)	7.03 (0.85)
**SENDER-RELATED PRONOUN-NOUN PAIRS**			
Valence	3.40 (0.83)	3.64 (0.81)	6.67 (1.35)
Arousal	4.16 (1.47)	4.09 (1.70)	4.31 (1.73)
Self-Relevance	2.91 (1.51)	3.11 (1.48)	3.96 (1.82)

*Scales ranged from 1 (extremely negative valence, extremely low arousal, extremely low self-relevance) to 9 (extremely positive valence, extremely high arousal, extremely high self-relevance). Standard deviations are in parentheses*.

***Manipulation check***. None of the participants reported consistently using any strategy throughout the experiment. Some participants (*N* = 14) reported that they repeated the labels during face processing, but retrospectively none of them had the impression that this had reduced the emotionality of the faces or their own feeling state. Subjects reported no difficulties in understanding the intention of the labels, relating them spontaneously to either the self or the sender's face.

### Experiment 2—labeling with intentional regulation instructions

#### Electrophysiological data

***Affective Labels***. P1, N1, and EPN amplitude modulations did not differ between self- and sender-related labels. However, akin to experiment 1, LPP amplitudes were more pronounced for self-related than for sender-related affective labels, *condition: F*_(1, 16)_ = 7.08, *p* = 0.02.

***Faces***. In the P1 time-window a main effect of *condition* was observed, *F*_(2, 32)_ = 3.7, *p* = 0.041. Amplitudes of P1 were significantly enhanced for emotional faces during intentional regulation as compared to passive face processing, especially when using self-related labels for emotion regulation, *self*: *F*_(1, 16)_ = 6.23, *p* = 0.023, *sender*: *F*_(1, 16)_ = 3.68, *p* = 0.073. During passive viewing, P1 amplitudes did not differ between emotional and neutral faces, *F*_(3, 48)_ = 1.8, *p* = 1.7.

In the N170 time-window, the factor *condition* showed only a trend toward significance, *F*_(2, 32)_ = 2.8, *p* = 0.079, which indicated a slight reduction in N170 amplitudes to emotional faces during emotion regulation trials relative to passive viewing of emotional faces. During passive viewing, N170 amplitudes were more pronounced for emotional than for neutral faces, especially for fearful faces, *F*_(1, 16)_ = 6.02, *p* = 0.026. The factor *valence* was significant, *F*_(3, 48)_ = 4.68, *p* = 0.046.

In the EPN time-window a main effect of *emotion* was observed, *F*_(2, 32)_ = 3.9, *p* = 0.037: EPN amplitudes were more pronounced for fearful and angry faces than for happy faces, *F*_(1, 16)_ = 4.94, *p* = 0.041; *F*_(1, 16)_ = 5.0, *p* = 0.04. There was no significant interaction of the factors *emotion x condition*, *F*_(4, 64)_ = 0.67, *p* = 0.61. During passive viewing, EPN amplitudes did not differ significantly between emotional and neutral faces. The factor *valence* was not significant, *F*_(3, 48)_ = 3.48, *p* = 0.18.

In the LPP/slow wave time-window amplitudes were modulated by the main factor *condition*, *F*_(2, 32)_ = 5.1, *p* = 0.021. Amplitudes were significantly greater for emotional faces during the “label own emotion” condition compared to the passive viewing condition, *F*_(1, 16)_ = 10.58, *p* = 0.005, and also compared to the “label sender's emotion” condition, *F*_(1, 16)_ = 7.0, *p* = 0.018. During “label sender's emotion”, amplitudes were attenuated compared to passive viewing of emotional faces. However, this attenuation for emotional faces during “label sender's emotion” was only significant when LPP amplitudes elicited during “label sender's emotion” were compared to passive viewing of neutral faces.

Akin to experiment 1, no significant differences were observed for ERP latencies.

**Figure 3:**
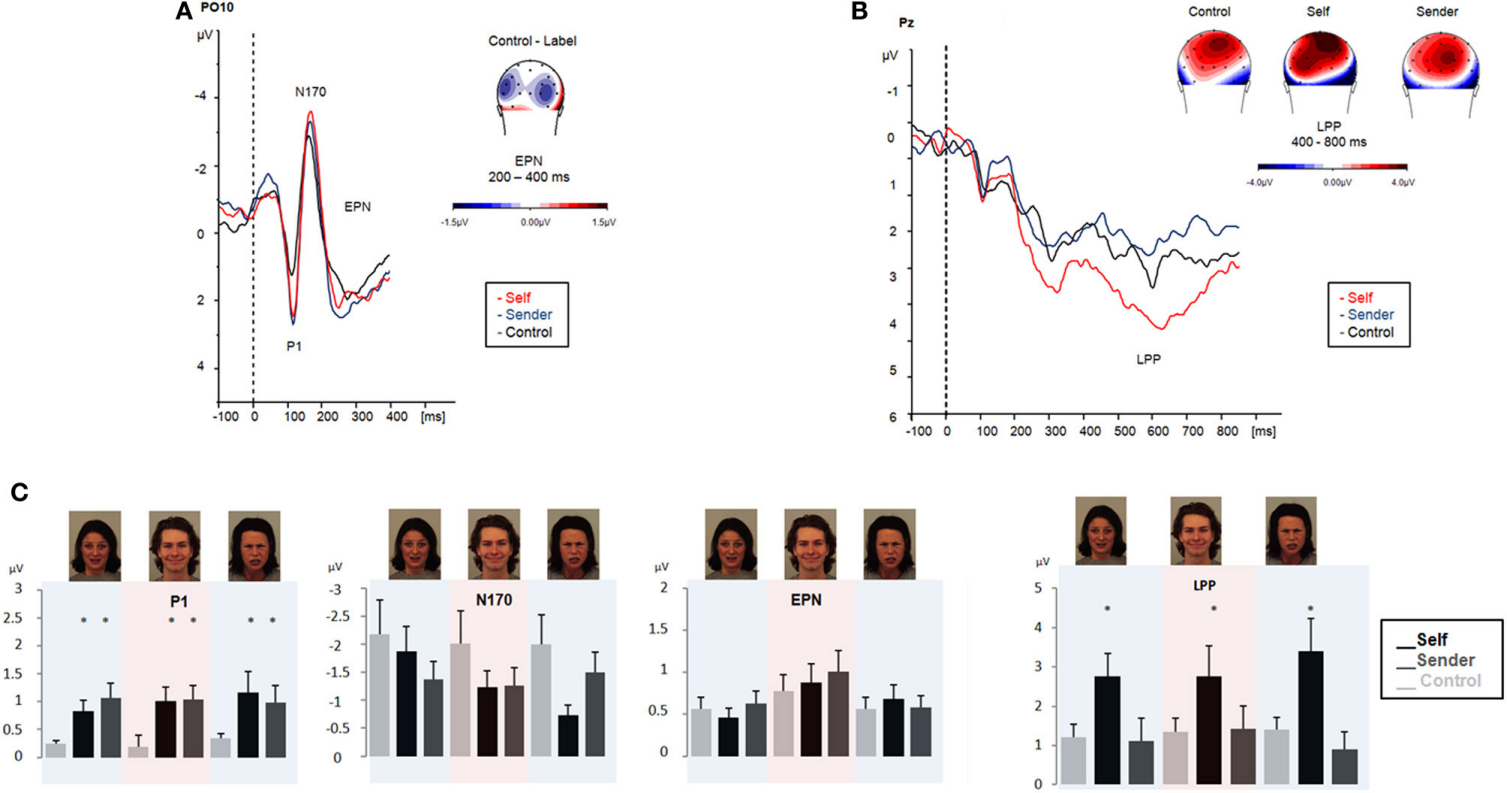
**ERPs obtained during emotional face processing in experiment 2 (affect labeling with intentional regulation instruction)**. Passive viewing of emotional faces (black line). Upper left panel **(A)** displays ERP modulation patterns in the early time-windows (P1, N170, and EPN, respectively). Upper right panel **(B)** displays amplitude modulation of the LPP. Maps display the topographic distribution of the ERPs in μV. Lower panel **(C)**: overview of P1, N170, EPN and LPP modulation (means and SEMs) during emotional face processing in experiment 2. Color shadings highlight ERP modulation for each emotional category (fearful, angry, and happy).

**Figure 4:**
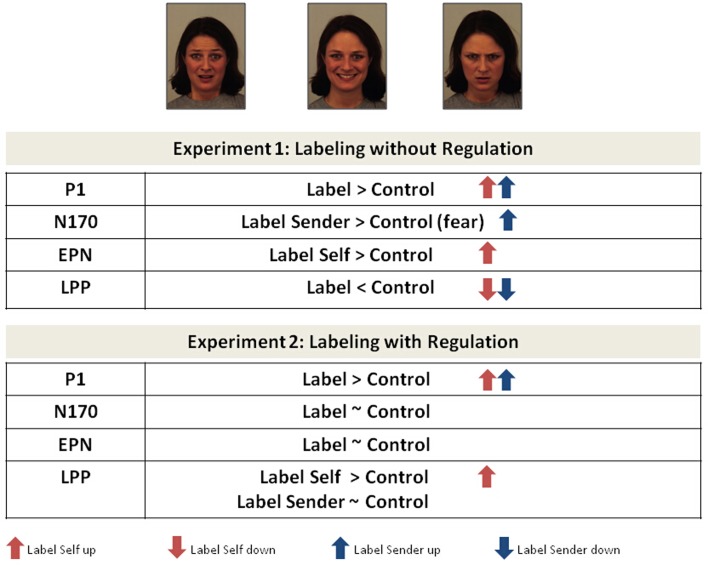
**Modulation of event-related brain potentials obtained during experiment 1 and experiment 2**. Arrows indicate the direction of the modulation (up vs. down) compared to passive face processing. Only significant results are considered, trends are reported in the text.

ERP results of experiment 2 are displayed in Figure 3. Results obtained from both experiments are summarized in Figure 4.

#### Behavioral data

***Ratings***. Fearful and angry faces were rated as significantly more negative in valence compared to happy faces, *F*_(2, 32)_ = 129.6, *p* < 0.01. This was true regardless of whether faces were cued with affective labels or presented during the control condition. Valence ratings also differed between emotional faces and neutral faces (see Table 3). Again, this was true regardless of the condition in which the faces had been presented during the experiment. Arousal ratings were significantly modulated by the factor *condition*, *F*_(2, 32)_ = 7.07, *p* < 0.01. Emotional faces presented in the “label own emotion” condition were retrospectively rated higher in arousal than faces being presented in the control condition of passive viewing and the “label sender's emotion” condition.

**Table 3:** **Experiment 2: rating data of emotional faces for the faces that were preceded by self-related pronoun-noun pairs, sender-related pronoun-noun pairs, or no pronoun-noun pairs (control condition)**.

	**Fear**	**Anger**	**Happiness**	**Neutral**
**SELF-RELATED**				
Valence	3.22 (0.91)	3.02 (1.00)	6.54 (1.15)	
Arousal	6.02 (1.56)	5.97 (1.79)	5.00 (1.26)	
**SENDER-RELATED**				
Valence	3.37 (0.89)	3.12 (1.11)	6.72 (1.11)	
Arousal	5.56 (1.48)	5.74 (1.30)	4.89 (1.49)	
**CONTROLS**				
Valence	3.32 (1.14)	3.14 (1.23)	6.44 (1.02)	4.69 (0.94)
Arousal	5.34 (1.40)	5.83 (1.32)	4.72 (1.48)	3.41 (1.67)

*Neutral faces were presented during the control condition only*.

*Scales ranged from 1 (extremely negative valence, extremely low arousal) to 9 (extremely positive valence, extremely high arousal). Standard deviations are in parentheses*.

Self-related pronouns were rated higher in arousal and in self-relevance than pronouns related to the sender, *arousal: F*_(1, 16)_ = 14.2, *p* < 0.01, *self-relevance*: *F*_(1, 16)_ = 37.0, *p* < 0.01. Positive pronoun-noun pairs were rated significantly higher in valence compared to pronoun-noun pairs describing fear and anger, as was indicated by a significant main effect of *emotion*, *F*_(2, 32)_ = 129.1, *p* < 0.01. A significant interaction of *emotion x condition*, *F*_(2, 32)_ = 12.1, *p* < 0.01 revealed a self-positivity bias: akin to experiment 1, valence ratings were higher for positive pronoun-noun pairs when related to the self than when related to the sender.

Rating data are summarized in Table 3 and Table 4.

**Table 4:** **Experiment1: mean valence, arousal, and self-relevance ratings of self-related and sender related pronoun-noun pairs obtained after the experimental recordings**.

	**Fear**	**Anger**	**Happiness**
**SELF-RELATED PRONOUN-NOUN PAIRS**			
Valence	2.82 (0.91)	3.43 (0.84)	7.56 (0.72)
Arousal	5.09 (1.87)	4.79 (1.74)	6.16 (1.40)
Self-Relevance	5.99 (1.56)	6.08 (1.18)	7.33 (0.88)
**SENDER-RELATED PRONOUN-NOUN PAIRS**			
Valence	3.53 (1.02)	3.79 (0.86)	6.74 (0.95)
Arousal	3.94 (1.87)	3.95 (1.84)	4.81 (1.89)
Self-Relevance	3.21 (1.38)	3.09 (1.43)	4.02 (1.85)

*Scales ranged from 1 (extremely negative valence, extremely low arousal, extremely low self-relevance) to 9 (extremely positive valence, extremely high arousal, extremely high self-relevance). Standard deviations are in parentheses*.

***Manipulation check***. All participants were familiar with the task prior to the start of the experiment. All but 2 of them reported using a particular strategy to control their feelings when labeling their own or the sender's emotion. As did the participants in experiment 1, they reported rehearsing the labels during face processing. However, in contrast to the participants in experiment 1, they reported rehearsing the labels to experience the faces more intensively, for instance by linking faces with an autobiographical event in the “label own emotion” condition. During the “label sender's emotion” condition most subjects reported rehearsing the cues to increase the emotional distance between the self and the sender's face (*N* = 10 subjects). Some (*N* = 6) additionally tried to not show any feelings at all (emotion suppression) or to not empathize with the sender. Participants reported no difficulties in relating the cues to their own emotion or the emotion of the face. Furthermore, they reported that their feelings had increased during the “label own emotion” blocks and had the opposite impression in the “label sender's emotion” blocks. Ratings obtained after each block also indicated that subjects found it somewhat harder to regulate their emotions during the “label sender's emotion” blocks compared to during the “label own emotion” blocks. ANOVAs revealed a trend for the main factor *condition*, *F*_(1, 16)_ = 3.5, *p* = 0.07. In addition, a significant main effect of *emotion*, *F*_(2, 32)_ = 3.6, *p* = 0.038, indicated that amongst the to be regulated emotions (fear, anger, happiness), participants had the impression of putting more effort on the regulation of fear. Regarding the direction of their success, they reported that feelings became more positive, particularly when viewing happy faces and during the “label own emotion” blocks. This was indicated by a significant main effect of the factor *emotion*, *F*_(2, 32)_ = 27.76, *p* < 0.01, and a significant interaction of the factors *emotion* x *condition*, *F*_(2, 32)_ = 8.56, *p* = 0.001.

### Correlation analysis of ERPs and self-report measures

In experiment 1, correlation analyses revealed no significant results. Neither in the early nor in the late face processing time-windows about 1 s a significant relationship between ERPs and any of the selected self-report indices was found. In experiment 2, amplitudes of the slow wave showed a positive correlation with self-esteem (Pearson's *r* = 0.4, *p* = 0.035) during the “label own emotion” regulation blocks, and a significant negative correlation with empathy (Pearson's *r* = −0.6, *p* = 0.004) during the “label sender's emotion” regulation blocks. Emotional intelligence was also negatively correlated with the amplitude of the slow wave during the “label sender's emotion” regulation (Pearson's *r* = −0.43, *p* = 0.04). However, when *p*-values were Bonferroni corrected for multiple comparisons (*p* = 0.008), only for empathy was the correlation still significant.

## Discussion

Two separate experiments were conducted to investigate the impact of affective labels on face processing during spontaneous, automatic emotion processing or in an intentional emotion regulation context. EEG-ERP methodology was used to examine how decoding emotions from facial expressions changes when faces are preceded by verbal labels that vary in the extent to which they describe one's own emotion or the emotion expressed by the sender.

Processing of self- and sender-related affective labels increased perceptual processing of emotional faces as early as in the P1 time-window. The P1 component is assumed to reflect a global and coarse processing of facial stimulus features in the primary visual cortex. This stage temporally precedes a more fine grained, configural analysis of the structural features of the face, this later stage being reflected in amplitude modulations of the face specific N170 component (e.g., Bentin et al., 1996). Modulation of the P1 component by emotions and context has been reported in some but not all face processing studies (for an overview Righart and de Gelder, 2006). However, agreement exists that very early facial feature processing as reflected by the P1 can be modulated by task-related and context-dependent top-down processes (Heinze et al., 1994; Rauss et al., 2012). Processing of affective labels could influence early facial feature processing via top-down cognitive mechanisms of anticipation, or by activating conceptual processing of emotional faces in anticipation of their encounter. During passive viewing, faces were cued by letter strings instead of affective labels, but P1 amplitudes did not differ between emotional and neutral face conditions. Due to their semantic content, words as labels possess a greater anticipatory signal character than meaningless letter strings. P1 modulation by self- and sender-related affective labels occurred independently of the emotional valence of the faces (happy, fearful, or angry) and across experiments (automatic processing in experiment 1 vs. intentional emotion regulation in experiment 2), supporting the robustness of this observation.

The perceptual processing stages that followed the P1 and which were indexed by the N170 and the EPN component were influenced differently by self- and sender-related labels. As predicted, structural encoding of fearful faces (N170) increased significantly when verbal labels described the emotion of the sender's face, whereas cueing the faces with self-related labels describing the reader's own emotion facilitated attention capture by emotional faces in the time-window of the EPN component, after the N170. In contrast to the N170, the EPN is considered to reflect early conceptual and semantic analysis of a stimulus in the ventral visual processing stream (Schupp et al., 2006; Kissler et al., 2007). The N170 and the EPN effects were significant in experiment 1 and not observed in experiment 2, supporting their unintentional and implicit nature.

This corroborates the idea that “what” is to be labeled during affect labeling and “how” this is done (automatically or intentionally) influences both the direction and the intensity of our perceptual experiences. Decoding emotions from facial expressions is indeed not fully determined by the sensory information derived from the face, nor is it completely insensitive to contextual factors or independent from the experience on the perceiver's side. Also, the relationship between language and perception is stronger than traditionally assumed (Lindquist and Gendron, 2013). Not only does reading emotion words activate our sensory and motor systems, activation in these systems is also temporarily reduced when access to emotion concepts is blocked experimentally (Lindquist et al., 2006; Gendron et al., 2012) or when concept activation during face processing is changed by verbal negation (Herbert et al., 2012). The present observations, including P1, N170, and EPN modulations by affective labels, further emphasize an embodied view of language. They demonstrate that even minor linguistic variations that change the personal reference or ownership of a particular emotion concept can be powerful mediators of emotion perception. While spontaneous processing of affective labels describing one's own emotions increases the motivational relevance of emotional faces, be they happy, angry, or fearful (see EPN results), sender-related labels seem to improve emotion recognition more specifically by facilitating decoding of structural information from the face, especially from fearful faces (see N170 results).

Processing of affective labels modulated later stages of face processing as well. Again, results differed between the two experiments, supporting the notion of psychologically and physiologically different mechanisms underlying automatic and intentional affect labeling. Spontaneous processing of affective labels reduced the amplitude of the late positive potential (LPP) to fearful, happy, and angry faces relative to the control condition of passive viewing. Moreover, this was observed for emotional faces cued by self- and sender-related affective labels. The LPP and the slow wave are cortical correlates of sustained attention and depth of stimulus encoding (Kok, 1997; Schupp et al., 2000; Moratti et al., 2011). Lower LPP amplitudes thus imply a reduction in depth of stimulus processing during automatic and hence unintentional affect labeling. This was not restricted to the processing of fearful or angry faces, applying to all faces expressing negative emotions. To the contrary, processing of self- and sender-related labels seemed to reduce processing costs for both, negative and positive emotional facial expressions.

A reduction in cortical processing depth, however, does not necessarily imply a dampening of affect on a subjective experiential level. When asked post-experimentally, none of the participants in experiment 1 had the impression that processing of affective labels had transiently dampened the own feeling state during the experiment. Ratings obtained for a subset of the faces after the experiment also indicated no changes in perceived valence or arousal. This underscores findings from previous studies suggesting that processing of affective labels has incidental effects on a bio-physiological level, but not necessarily on a subjective experiential level (Kircanski et al., 2012).

During active emotion regulation a different pattern emerged, distinguishing between automatic and intentional affect labeling processes. Unlike in experiment 1, amplitudes of the LPP/cortical slow wave in experiment 2 were enhanced for emotional faces when subjects used the self-related affective labels for emotion regulation during face processing. Likewise, they reported the feeling that the emotionality of the faces increased during the “label own emotion” regulation blocks and rated the faces afterwards as higher in arousal than the emotional faces that had been presented in the “label sender's emotion” or the passive viewing conditions. Increased processing in the “label own emotion” regulation condition contrasts with the view that making oneself aware of one's own emotions would transiently dampen the feeling state itself. It also runs counter to the assumption that affect labeling would always have a down-regulatory effect on emotional stimulus processing (Lieberman, 2007, 2011), independent of the personal reference properties of the label and the participants' intentions.

The results of experiment 2 suggest that intentional affect labeling can intensify encoding of emotional faces and make their content more intense and self-relevant. This is in line with observations from imaging studies on self-referential processing of emotional stimuli. Self referential processing of emotional stimuli activates medial prefrontal cortex regions, which are part of the salience network (Schmitz and Johnson, 2007). Parts of this network have been found to be active during reading of self-related emotional pronoun-noun pairs as well (Herbert et al., 2011c). In line with these observations, self-related emotional pronoun-noun pairs elicited larger LPP amplitudes relative to sender-related emotional-pronoun-noun pairs, which corroborates findings from recent studies showing similar effects (e.g., Walla et al., 2008; Zhou et al., 2010; Herbert et al., 2011a,b; Shi et al., 2011) and supports the notion that participants discriminated between the self and the other during reading of self- and sender-related labels.

Experiencing a strong sense of ownership can diminish self-other boundaries, such as when watching one's own face and the face of a sender being touched simultaneously (Maister et al., 2013). Labeling one's own emotion seems to provide another way to get in touch with one's own emotion while viewing someone else's face, particularly when there is the intention to do so, as seen in experiment 2. This could have effects similar to the resonance of being touched in real time as it might help synchronizing one's own feelings with the expressions of the sender's face.

Resonance between sender and observer might also play a role when attempting to regulate one's own emotion by labeling the emotion of the sender's face. In the present study, empathy and emotional intelligence were inversely related with amplitudes of the slow wave during the “label sender's emotion” conditions. In addition, self-esteem, which reflects the most fundamental appraisals about the self, was positively correlated with depth of face processing during the “label own emotion” blocks, corroborating theoretical conceptions that link self esteem with improved self consciousness or an improved self-awareness (Branden, 1969). Positive correlations between the personality measures found here might reverse when, instead of healthy subjects, clinically relevant samples with poor self-esteem, low empathy, and high emotional blindness are investigated. Gender likewise could play a role because more females than males took part in the present experiments. In any event, inter-individual differences should be taken into account in future research. In the present study, results on inter-individual differences can only be considered tentative due to their exploratory nature and the small sample sizes studied.

It has been debated to what extent interventions in which individuals are asked to focus on their own emotion and to become aware of them are helpful for emotion regulation. Looking at clinical disorders, internal self focus of attention can give rise to negative feelings of distress and heightened physiological arousal (Ingram, 1990). Similar observations of an increase in symptomatology (e.g., increase in distress, negative mood, and physical symptoms) have been reported immediately after expressive writing (Pennebaker and Beall, 1986; Pennebaker and Chung, 2007, 2011). In the present study, participants reported an increase in feelings during intentional affect labeling as well. However, this increase in feelings when labeling one's own emotion did not push subject's mood in a negative direction. A major difference between self monitoring in clinical disorders, expressive writing, and intentional affect labeling is that, during intentional affect labeling, labels provide a concrete context for appraising one's feelings and concomitant bodily changes, the latter often being verbally accessible only along simple physical dimensions of valence (good-bad) and arousal (calm-arousing). In this sense, using self- and sender-related labels actively and intentionally for emotion regulation seems to have comparable effects on emotion processing and cognitive reappraisal. A more speculative possibility is that using self- and sender-related labels actively and intentionally for emotion regulation might have facilitated verbal self-guidance and self-regulation by means of inner speech (Morin, 2005). Future studies could test this assumption.

## Conclusion

Language has long been considered as being somewhat independent from emotions, both with regard to its capacity to induce emotions and with regard to its potential to up- and down-regulate emotion processing in accord with situational demands. The present study sheds light on the mechanisms underlying the emotional regulatory capacity of language. The results are the first to show that “what” is being labeled verbally (e.g., one's own emotion or the emotion of the sender's face) during face processing and how intentionally this is done matters. The present observations therefore pave the way for a differentiated view of language as a context for emotion processing. Some of our observations are specifically suggestive for future research, particularly the observation, that, with few exceptions, processing of affective labels with or without regulatory intent did not interact with the emotional valence of the faces, i.e., regardless of whether facial expressions were fearful, angry, or happy. That is, ERP modulations reflected the different experimental conditions of self vs. sender labeling, rather than the emotional content itself. Previous studies can provide limited information on this issue because stimulus material was limited to either fearful or angry or stereotypic material, whereas other researchers used emotional pictures instead of faces as stimuli. Similarly, much of the previous research outlined in this paper used functional imaging methods while the present studies delineate the influence of affective labels on face processing in real time with a resolution of milliseconds, by means of EEG methodology. Future studies using larger numbers of stimuli or different sets of stimuli, including emotion pictures or voices, are needed to test the valence specificity assumption. Inclusion of different stimulus materials besides faces will also show if effects occur across sensory modalities, which could improve our understanding of language-emotion-cognition interactions in real life social situations.

### Conflict of interest statement

The authors declare that the research was conducted in the absence of any commercial or financial relationships that could be construed as a potential conflict of interest.
